# A Clinical Decision Support Engine Based on a National Medication Repository for the Detection of Potential Duplicate Medications: Design and Evaluation

**DOI:** 10.2196/medinform.9064

**Published:** 2018-01-19

**Authors:** Cheng-Yi Yang, Yu-Sheng Lo, Ray-Jade Chen, Chien-Tsai Liu

**Affiliations:** 1 Graduate Institute of Biomedical Informatics College of Medical Science and Technology Taipei Medical University Taipei Taiwan; 2 Department of Medical Informatics Industrial Technology Research Institute Hsinchu Taiwan; 3 Department of Surgery School of Medicine College of Medicine, Taipei Medical University Taipei Taiwan; 4 Taipei Medical University Hospital Taipei Taiwan

**Keywords:** duplicate medication, adverse drug reaction, clinical decision support system, PharmaCloud

## Abstract

**Background:**

A computerized physician order entry (CPOE) system combined with a clinical decision support system can reduce duplication of medications and thus adverse drug reactions. However, without infrastructure that supports patients’ integrated medication history across health care facilities nationwide, duplication of medication can still occur. In Taiwan, the National Health Insurance Administration has implemented a national medication repository and Web-based query system known as the PharmaCloud, which allows physicians to access their patients’ medication records prescribed by different health care facilities across Taiwan.

**Objective:**

This study aimed to develop a scalable, flexible, and thematic design-based clinical decision support (CDS) engine, which integrates a national medication repository to support CPOE systems in the detection of potential duplication of medication across health care facilities, as well as to analyze its impact on clinical encounters.

**Methods:**

A CDS engine was developed that can download patients’ up-to-date medication history from the PharmaCloud and support a CPOE system in the detection of potential duplicate medications. When prescribing a medication order using the CPOE system, a physician receives an alert if there is a potential duplicate medication. To investigate the impact of the CDS engine on clinical encounters in outpatient services, a clinical encounter log was created to collect information about time, prescribed drugs, and physicians’ responses to handling the alerts for each encounter.

**Results:**

The CDS engine was installed in a teaching affiliate hospital, and the clinical encounter log collected information for 3 months, during which a total of 178,300 prescriptions were prescribed in the outpatient departments. In all, 43,844/178,300 (24.59%) patients signed the PharmaCloud consent form allowing their physicians to access their medication history in the PharmaCloud. The rate of duplicate medication was 5.83% (1843/31,614) of prescriptions. When prescribing using the CDS engine, the median encounter time was 4.3 (IQR 2.3-7.3) min, longer than that without using the CDS engine (median 3.6, IQR 2.0-6.3 min). From the physicians’ responses, we found that 42.06% (1908/4536) of the potential duplicate medications were recognized by the physicians and the medication orders were canceled.

**Conclusions:**

The CDS engine could easily extend functions for detection of adverse drug reactions when more and more electronic health record systems are adopted. Moreover, the CDS engine can retrieve more updated and completed medication histories in the PharmaCloud, so it can have better performance for detection of duplicate medications. Although our CDS engine approach could enhance medication safety, it would make for a longer encounter time. This problem can be mitigated by careful evaluation of adopted solutions for implementation of the CDS engine. The successful key component of a CDS engine is the completeness of the patient’s medication history, thus further research to assess the factors in increasing the PharmaCloud consent rate is required.

## Introduction

Duplication of medication can be defined as a patient being prescribed more than two medications of the same therapeutic class (including different doses, forms, frequencies, or routes) within an overlapping period, with one of the prescriptions being clinically redundant [1-3]. The duplication of medication orders is a critical issue that can result in some patients being affected by adverse drug reactions (ADRs) [4-6]. The potential for duplication of medications has increased, with patients visiting a greater number of different hospitals and following more extensive medication regimens. This issue particularly affects elderly patients and those suffering from chronic diseases [7-9]. A previous study indicated that physicians and pharmacists can help reduce unnecessary prescriptions and optimize a patient’s drug therapy regimen by examining a patient’s full medication record [1]. Reducing duplicate medications and treatment can contribute significantly to preventing ADRs.

In addition, duplication of medications increases overall medical expenditures [4-6], causes serious environmental pollution, and wastes medical and social resources [10,11]. Each year in Taiwan, more than 3 tons of prescribed medications go unused [11]. A study also indicated that 8.8% of outpatients received duplicate medications across different health care facilities in Japan [12]. In the United Kingdom, approximately £300 million of medicines prescribed by the National Health Service are wasted each year [10]. The issue of duplicate medications and its impact on patient safety have received attention in several countries.

More and more information and communication technologies, such as clinical decision support systems (CDSSs), have been proposed as a solution for improving medication safety. Many studies have suggested that a computerized physician order entry (CPOE) system combined with a CDSS could help in preventing ADRs [5,6,13], thereby reducing medication expenditure [14]. However, even when using such a system, duplication of medication can still occur due to a lack of an infrastructure supporting the integration of patients’ medication records prescribed by different health care facilities in general, including clinics, doctor offices, medical centers, or large hospitals. Thus, when a patient is transferred from one hospital to another, or visits more than one hospital for the same condition, physicians may not be aware of the medication prescribed at other hospitals and may prescribe duplicate medications despite using a CPOE system with a CDSS.

In Taiwan, the National Health Insurance Administration (NHIA) has implemented approaches for sharing patients’ medical care information nationwide, including health smart cards [4] and a Web-based medication query system based on a national medication repository known as the PharmaCloud [4,15]. The PharmaCloud contains the most complete and up-to-date version of a patient’s medication history. According to NHIA policy, health care facilities must upload a patient’s prescribed medications to the PharmaCloud within 24 hours after the patient’s visit [15,16].

Currently, the PharmaCloud stores the latest 3 months of each patient’s prescribed medication records. It supports two access modes for authorized clinical professionals. One is an online query through a Web browser interface; the other is a batch download. By using the online query mode, an authorized physician can access patients’ medication histories in the PharmaCloud through a Web browser. When the physician wants to prescribe medication orders for a patient, he or she can use the Web browser to submit queries about the patient’s medication history prior to ordering a prescription. A physician can check that information on the browser manually and then use that information to make independent decisions about the prescription to avoid prescribing duplicate medications. Most health care facilities encourage their physicians to use the online query mode to access the PharmaCloud. However, physicians are usually very busy and so this approach may not be feasible.

In the batch download mode, a patient’s medication history can be downloaded from the PharmaCloud provided the patient has signed an informed consent form allowing the authorized physicians access and has made an appointment at least one day in advance. We will refer to the informed consent form as the PharmaCloud consent form. In this approach, the downloaded patients’ medication histories have to be integrated into a CPOE system so that the CPOE can verify the prescription to see if there is any potential duplicate medication and other ADRs. However, CPOE systems are complex because they must access data from various systems within a hospital. Furthermore, electronic health record (EHR) systems are usually adopted incrementally [17,18]. Thus, a new approach to design a flexible and scalable decision support system that integrates the PharmaCloud and a CPOE system to prevent duplicate medications and other ADR events is needed.

In this study, we developed a modularized clinical decision support (CDS) engine that can support duplicate medication checks based on the PharmaCloud. We also analyzed the impact of the CDS engine on patient encounter time and physicians’ responses to handling potential duplicate medication alerts. These results could provide insights to adopt the CDS engine and recommendations to improve the efficiency in medication safety checks.

## Methods

### Settings

For this study, the CDS engine was developed and installed at Taipei Medical University Hospital, a teaching hospital with nearly 800 beds. The hospital has a highly informative infrastructure and is a certified Healthcare Information and Management Systems Society EHR Adoption Model stage 6 hospital [19]. At this hospital, the backend repositories and databases of CPOE, online registration/appointment, and drug information management systems have been integrated. Although a CDS engine may perform many decision support functions, at this stage the implemented CDS engine supported the function of duplicate medication checking only.

### Framework of the Clinical Decision Support Engine and its Interactions With the PharmaCloud and Computerized Physician Order Entry Systems

**Figure 1:**
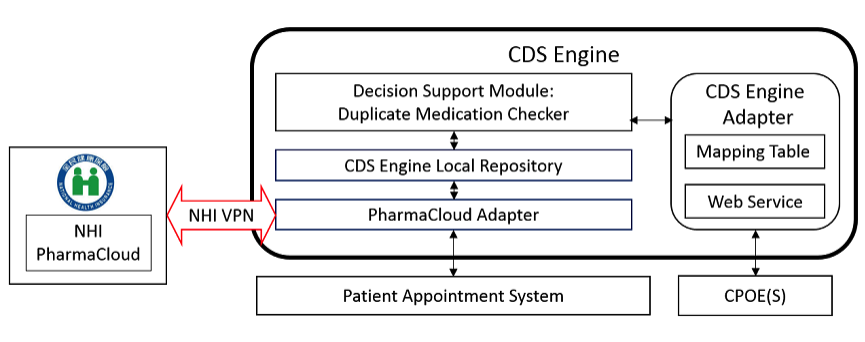
The integrated computerized physician order entry (CPOE) system and clinical decision support (CDS) engine for detecting potential duplicate medications. NHI: National Health Insurance; VPN: virtual private network.

The framework of the CDS engine and interactions with the PharmaCloud and a CPOE system are presented in Figure 1. The implemented CDS engine consisted of four major components: the PharmaCloud adapter, CDS engine local repository, the duplicate medication checker, and the CDS engine adapter as described subsequently.

#### The PharmaCloud Adapter

The PharmaCloud adapter is used to access a patient’s visit appointment information registered in the patient appointment system and to verify whether the patient signed the PharmaCloud consent form for PharmaCloud access. If so, the PharmaCloud adapter retrieves the patient’s last 3 months of medication records from the PharmaCloud via batch download over the National Health Insurance (NHI) virtual private network (VPN).

#### Clinical Decision Support Engine Local Repository

The CDS engine local repository was implemented using the PostgreSQL relational database system [20] to store the patients’ medication history data retrieved from the PharmaCloud. The medication history contains all medication records prescribed in the last 3 months by the health care facilities in Taiwan. The medication record contains information including the Anatomical Therapeutic Chemical (ATC) Classification name, NHI drug code, drug ingredients, drug name, prescribing date, number of days it was prescribed for, and the number of days of drug treatment remaining.

#### Decision Support Module: Duplicate Medication Checker

The decision support module is a decoupled, thematic design approach that allows health care facilities to add, update, and delete customized medication verification modules (eg, duplicate medication, drug-drug interaction, and maximum dosage). The duplicate medication checker was one of the verification modules used in this study. It consists of a set of logic and rules for detecting duplicate medications. Duplicate medication is primarily identified using the ATC system [2,3]. We defined potential duplicate medication as two prescribed drugs (not necessary in the same prescription, but with an overlap between their start date and stop date) that had the same ATC level 4 codes (ie, the first four digits of the ATC codes are identical) [15,21-24]. The NHIA has released a cross-mapping table between NHI drug codes and ATC codes [15].

#### Clinical Decision Support Engine Adapter

The CDS engine adapter is an interface between the CDS engine and a CPOE system that allows the CPOE system to initiate the duplicate medication checker. It performs mapping functions between the hospital’s drug codes, NHI drug codes, and ATC codes. When a physician wants to prescribe a drug for a patient, the CPOE system sends the drug details, including the patient’s identification, drug code, start date, and stop date, to the duplicate medication checker via the CDS engine adapter, which then converts the drug details into a form that can be interpreted by the duplicate medication checker. After checking for duplicate medication, the duplicate medication checker returns the result to the CPOE system via the CDS engine adopter.

#### Extension of Decision Support Function

The CDS engine is different from the traditional CDSS coupled with CPOE. The CDS engine has a decoupled decision support module from the hard-coded rules in CPOE. The module provides a thematic decision rules design approach. Health care facilities are able to maintain several independent thematic CDS modules for different CDS applications. These independent configurable knowledge rule modules allow CPOE to invoke few configurations and decrease the code change of the original CPOE. The scalable and flexible nature of the framework facilitates health care facilities to integrate the CDS function into their existing CPOE system. The steps involved in the extension of the CDS engine rule module are as follows:

1. Defining the theme of the decision support module. In this study, we defined a duplicate medication checker to distinguish duplicate medications. The theme of the decision support module can be extended using a drool file [25]. Creating a new decision support module or modifying the original decision support module is possible in the CDS engine.
2. Defining the input and output parameters and the decision support logics in the decision support module. Health care facilities must select the input parameters from the CPOE and local repository for the decision support logics, and those that are to be returned to the CPOE. The design of the decision support logics may be based on relevant clinical guidelines, regulations, protocols, or medication knowledge.
3. Retrieving the EHRs from the local repository. In our study, the EHRs were retrieved from PharmaCloud through the PharmaCloud adapter. In other scenarios, health care facilities could add other EHR adapters to retrieve different EHR sources.
4. Adding a Web service path to the CDS engine adapter. A health care facility can add a Web service URL for CPOE to invoke the added decision support module.
5. CPOE has an AJAX [26] Web service call from the CDS engine adapter to invoke the decision support module in a CPOE textbox; thus, physicians are alerted when prescribing medications.

#### Information Security Framework

To ensure a certain level of safety in storing medical information, we adopted some information security assumptions for both the EHR repositories and CPOE, such as secure tunnel, access control, and privacy control protection. In the secure tunnel, as PharmaCloud is deployed in the NHI VPN environment, the CDS engine must access the PharmaCloud through the NHI VPN. In the access control, we must have both the physician’s Healthcare Certification Authority card and the patient’s health smart card simultaneously inserted into the card reader to verify that the physician has the authority to access the patient’s medication history. Finally, the patient must sign the PharmaCloud consent form before the CDS engine batch downloads their medication history; if not, the CDS engine would not retrieve the patient’s medication history.

### Workflow for Detecting Potential Duplicate Medication Across Health Care Facilities With the Clinical Decision Support Engine

Patients who wish to allow their physicians at a health care facility to access their medication history in the PharmaCloud must complete the PharmaCloud consent form and submit it to the health care facility. When a patient wants to visit a doctor, he or she makes an appointment and registers in advance by using the patient appointment system of the health facility. If the patient’s consent is in effect at the time of the visit, the CDS engine retrieves the patient’s medication history for the past 3 months from the PharmaCloud and stores it into the CDS engine local repository. To evaluate the impact of the CDS engine on an outpatient clinical encounter, a clinical encounter log iss created to collect information about the patient and physician, the start and end time of the clinical encounter, the drugs prescribed by the physician, and the physician’s responses to potential duplicate medication alerts, if any.

**Figure 2:**
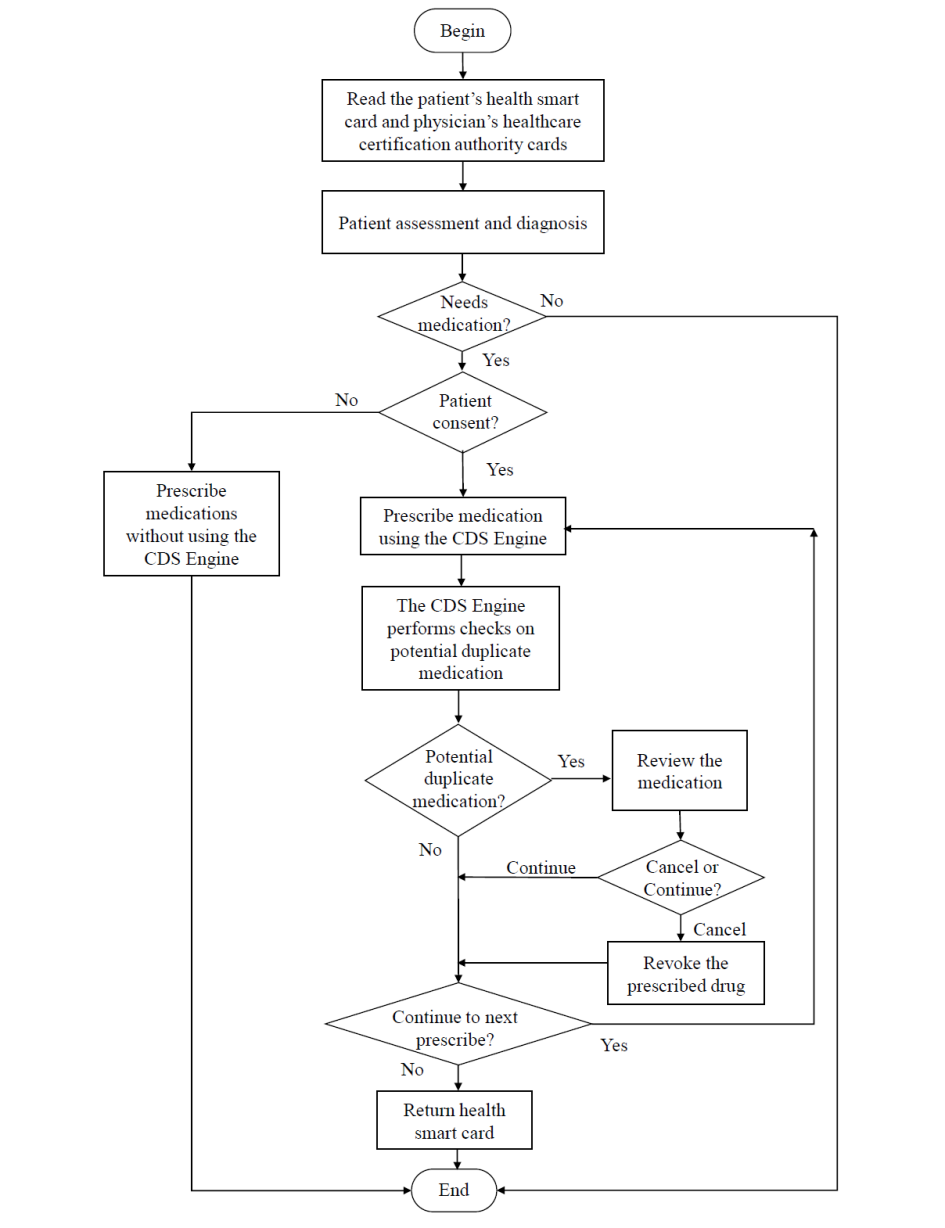
The prescription workflow using the clinical decision support (CDS) engine for detection of potential duplicate medication.

Figure 2 shows the prescription workflow of a clinical encounter using the CDS engine. First, the physician’s Healthcare Certification Authority card and the patient’s health smart card are simultaneously inserted into a card reader to initiate the clinical encounter. The CPOE system reads the physician’s and patient’s information. This information and the start time of the encounter are recorded into the clinical encounter log. The physician then conducts the patient assessment and diagnosis for the patient. If the patient does not require any medication, the workflow ends. If the patient requires medication, the physician prescribes a drug via the CPOE system. The CPOE system verifies whether the patient has signed the PharmaCloud consent form. If not, the physician simply uses the CPOE system to prescribe the drug without using the CDS engine. If the patient has signed the PharmaCloud consent form, the CPOE invokes the CDS engine duplicate medication checker to perform a duplicate medication check. The prescribed drug and the check result are also recorded into the clinical encounter log.

**Figure 3:**
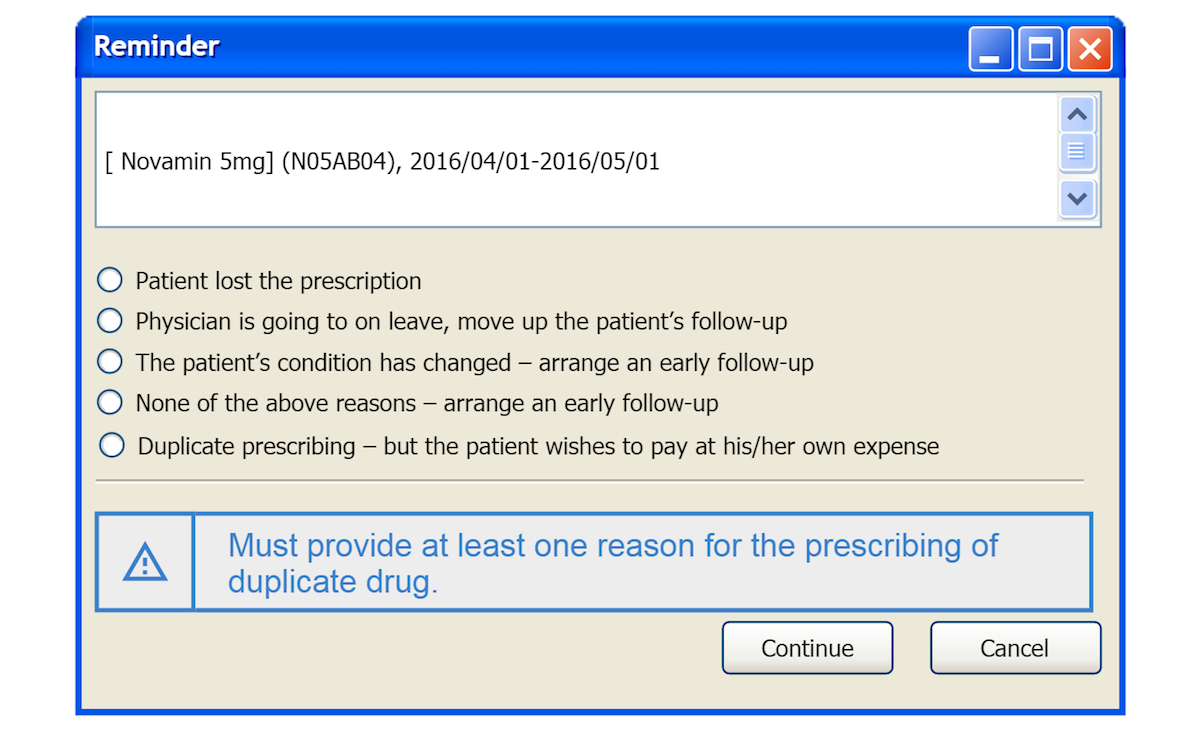
A screenshot of a pop-up screen showing an alert message that appears when a potential duplicate medication is detected. The screen presents the information about the duplicate drug (upper panel), response options for reasons for prescribing the medication (middle), and action to take (lower panel).

If the duplicate medication checker detects a potential duplicate medication (ie, the prescribed drug’s ATC level 4 code is the same as the one stored in the CDS engine local repository), it sends an alert to the CPOE system. The alert information, including drug name, ATC code, and start and stop dates, is then displayed on a pop-up screen (Figure 3, upper panel). Our hospital requires the physician to provide a reason for prescribing the duplicate drug in order to meet the NHI payment policy. Thus, the physician can click on one of the check buttons (Figure 3, middle) and then proceed to prescribe the subsequent drug by clicking on the “Continue” button. If the physician does not select a reason, he or she has to click on the “Cancel” button to revoke the prescribed drug. The reason for prescribing the duplicate drug and the physician’s response are recorded in the clinical encounter log. If no duplicate medication is found, the physician can continue prescribing drugs until no further drug prescription is required. Finally, the physician withdraws the patient’s health smart card from the card reader to end the clinical encounter. The ending time is also recorded in the clinical encounter log.

### Analysis of Impacts of the Clinical Decision Support Engine on Outpatient Services

The CDS engine has been integrated into a CPOE system of the hospital to support order entry processes, and this combined system has operated in four outpatient departments: medicine, surgery, gynecology-pediatrics, and “other” departments. The clinical encounter log was initiated to collect information about clinical encounters from April 1 to June 30, 2016. During this period, for each patient’s clinical encounter, the log collected the starting and ending time of the encounter, prescribed medication, and the physician’s response(s) to a potential duplicate medication alert, if any. The log could be used to analyze how the CDS engine affected the encounter time and to determine physicians’ responses to potential duplicate medication alerts in different outpatient departments.

To investigate clinical encounter time, we divided clinical encounters into two groups based on the clinical encounter log: “with CDS engine” and “without CDS engine.” Patients in the without CDS engine group were those who did not sign a PharmaCloud consent form; patients in the with CDS engine group were those who signed the PharmaCloud consent form. We also analyzed the characteristics of the consent rate, potential duplicate medication rate, and physicians’ response(s) to any potential duplicate medication alerts. We used the statistical software R version 3.3.1 [27] to perform a Wilcoxon rank sum test with a 5% level of significance to assess the difference in encounter time between the without CDS engine and the with CDS engine groups.

## Results

Overall, there were 178,300 patient visits to the four outpatient departments during the 3-month period, as shown in Table 1. There were 43,844 (24.59%) of patient visits in which the patients signed the PharmaCloud consent form, allowing their physicians to access their medication history stored in the PharmaCloud. That is, there were 43,844 and 134,456 patient visits in the with CDS engine and without CDS engine groups, respectively. In the without CDS engine group, there were 96,714 (71.93%) patient visits in which at least one medication order was prescribed. In the with CDS engine group, there were 31,614 (72.11%) patient visits in which at least one medication order was prescribed. Among these, 4227 (13.37%) prescriptions resulted in potential duplicate medication.

Table 2 shows the clinical encounter time in the with CDS engine and without CDS engine groups. A Wilcoxon rank sum test showed that the clinical encounter time in the with CDS engine group (median 4.3, IQR 2.3-7.3 min) was significantly longer than that in the without CDS engine group (median 3.6, IQR 2.0-6.3 min). A similar pattern was observed in the medicine, surgery, and other departments, but not in the gynecology-pediatrics department, where there was no significant difference between the groups. This might be because there are usually more medical checkups and procedures than medication treatments in gynecology-pediatrics outpatient services.

**Table 1:** Analysis of clinical encounters information during the 3-month data collection period.

Clinical encounters	With CDS^a^ engine (n=43,844)	Without CDS engine (n=134,456)	Total (N=178,300)
No medicine prescribed, n (%)	12,230 (27.89)	37,742 (28.07)	49,972 (28.03)
Medicine prescribed, n (%)	31,614 (72.11)	96,714 (71.93)	128,328 (71.97)
Potential duplicate medication, n	4227	—	
No potential duplicate medication, n	27,387	—	

^a^CDS: clinical decision support.

**Table 2:** Differences in clinical encounter time between with clinical decision support (CDS) engine and without CDS engine groups.

Departments	Without CDS engine			With CDS engine			*P*
	n	Median time in minutes, (IQR)	n		Median time in minutes, (IQR)		
Medicine	49,310	3.7 (2.2-6.2)	17,189		4.5 (2.6-7.4)	<.01	
Surgery	26,885	3.1 (1.5-5.8)	9031		3.7 (1.8-7.0)	<.01	
Gynecology-Pediatrics	9129	4.5 (2.5-7.7)	2209		4.6 (2.3-8.0)	.92	
Other	11,390	3.8 (2.2-6.4)	3185		4.3 (2.4-7.2)	<.01	
Total	96,714	3.6 (2.0-6.3)	31,614		4.3 (2.3-7.3)	<.01	

**Table 3:** Physicians’ responses to potential duplicate medications.

Department	Physician response, n (%)						
	Cancel^a^	Lost prescription^b^	Physician on leave^c^	Condition change^d^	Other^e^	Self-pay^f^	Total
Medicine	1049 (36.32)	87 (3.01)	91 (3.15)	905 (31.34)	528 (18.28)	228 (7.89)	2888 (100)
Surgery	603 (55.89)	38 (3.52)	9 (0.83)	226 (20.95)	155 (14.37)	48 (4.45)	1079 (100)
Gynecology-Pediatrics	88 (40.18)	11 (5.02)	0 (0)	88 (40.18)	16 (7.31)	16 (7.31)	219 (100)
Other	168 (48.00)	5 (1.43)	5 (1.43)	82 (23.43)	21 (6.00)	69 (19.71)	350 (100)
Total	1908 (42.06)	141 (3.11)	105 (2.31)	1301 (28.68)	720 (15.87)	361 (7.96)	4536 (100)

^a^Cancel: confirmed as a duplicate drug—cancel this drug.

^2^Lost prescription: the patient lost the prescription.

^c^Physician on leave: the physician is going on leave, plan earlier patient follow-up.

^d^Condition change: the patient’s condition has changed—arrange an early follow-up.

^e^Other: none of the above reasons—arrange an early follow-up.

^f^Self-pay: duplicate prescribing, but the patient wishes to pay at his or her own expense.

An alert was trigged by a drug in a prescription if it was detected as a potential duplicate medication. Among the 4227 potential duplicate medication prescriptions (Table 1, 13.37% of 31,614 prescriptions), more than two potential duplicate medication alerts occurred for 170 (totaling 479 potential duplicate medication alerts); therefore, a total of 4536 potential duplicate medication alerts were responded to by physicians in Table 3. In summary, 42.06 % (1908) of the alerts led to cancelation of the duplicate drugs to be prescribed (ie, clicked “Cancel” button). The remaining 2628 alerts did not lead to a cancelation response and were issued for the following reasons: 28.68% (1301) for “condition change” (the patient’s condition has changed—arrange an early follow-up), 15.87% (720) for “others” (none of the above reasons—arrange an early follow-up), 7.96% (361) for “self-pay” (duplicate prescribing, but the patient wishes to pay at his/her own expense for some reasonable reasons), 3.11% (141) for “lost prescription,” and 2.31% (105) for “physician on leave.” Notably, the most common reason for issuing duplicate drugs was “condition change.” The alerts enabled the physicians to review their prescriptions once again and, consequently, to prevent the duplication of medications.

In Table 4, the results show that 1843 prescriptions were confirmed as duplicate medication prescriptions from the 4227 potential duplicate medication prescriptions, or that 5.83% of prescriptions (1843/31,614) might result in duplicate medications.

**Table 4:** Prescriptions confirmed as duplicate medication prescriptions.

Department	Potential duplicate medication, n (%)	Canceled drug^a^, n	CDS^b^ engine prescription^c^, n	Canceled drugs/CDS engine prescriptions, %
Medicine	2685 (63.52)	1025	17,189	5.96
Surgery	1019 (24.11)	576	9031	6.38
Gynecology-Pediatrics	215 (5.09)	86	2209	3.89
Others	308 (7.29)	156	3185	4.90
Total	4227 (100)	1843	31,614	5.83

^a^The prescription had at least one drug confirmed as a duplicate drug and the doctor canceled this drug.

^b^CDS: clinical decision support.

^c^The prescription checked with the CDS engine.

## Discussion

### Principal Results

In our study, we developed a CDS engine to access the PharmaCloud (a national medication repository) to retrieve the medication records of patients from the previous 3 months. As per Taiwan NHI policies, health care facilities are required to upload their patients’ prescriptions within 24 hours after a visit. Thus, the CDS engine can access a fairly complete and up-to-date medication history. A previous study [22] showed that incomplete or delayed sharing of EHRs across health care facilities made it difficult for a CDSS to perform thorough checks of potential duplicate medications, resulting in a duplicate medication detection rate of 2.4%. Our approach increased the previously reported duplicate medication detection rate from 2.4% to 5.83% of total prescriptions. It shows that the more complete the medication history is, the better the protection from duplicate medication.

Nowadays, medication safety is a top priority for both patients and health care providers. However, it requires additional cost. Under our CDS engine framework, clinical encounter time was slightly (0.7 min) longer than when a CPOE system was used alone (from 3.6 min to 4.3 min) despite the ability to enhance medication safety. However, as we adopt more advanced and faster communication and computer technology to build CDS engines, the increased time can be mitigated. Thus, while implementing the CDS engine, to guarantee the desired level of medication safety, we should carefully evaluate the adopted solutions to meet the time requirements in clinical practice. Although the CDS engine takes an additional time of 0.7 min, it is still more efficient than the previous methods used in Taiwan [28]. Previously, CPOE invoked medication information stored in the health smart card to support medication decisions, but the prescription information was written by the physician for each patient, which required additional time (1.88 min) and resulted in more time utilized than when using the CDS engine with PharmaCloud.

Knowledge-based CDS generally offers two categories: “stand-alone CDS” and “CDS coupled with CPOE” [29]. The former is not directly integrated into the clinical workflow; a physician must enter patient information into both CPOE and CDSS, which can cause double data entry. The physician has to switch between the systems. Furthermore, it could not issue reminders when the physician prescribed medication using CPOE. Thus, this method is time-consuming and may compromise the clinical process, particularly during busy clinical practice. The latter one prevented double data entry and issued reminders when the physician prescribed medications. However, in the context of ever-improving and new medical knowledge, new clinical guidelines, regulations, policies, and EHRs (in general, due to limited budgets and resources, EHR systems are usually adopted incrementally by health care facilities [17,18]), CDS functions must be kept updated to prevent use of outdated knowledge [30]. Previous studies have shown that CDS rules are usually hard-coded or tight-bundled with CPOE or incorporated into CPOE [31,32]; thus, the CPOE program has to be updated once the rules are updated. This maintenance of rapidly changing knowledge and systems with complex rule sets can be expensive [29]. Therefore, we provided an innovative CDS engine and adopted the CDS engine to detect potential duplicate medication in this study. Firstly, the CDS engine provides a decoupled decision support module, a thematic decision rules design approach. Health care facilities can design or update these independent configurable CDS knowledge modules separately, such as potential duplicate medication checks, drug-drug interactions, and drug allergies. This would increase the scalability and extensibility of CDS with CPOE. Secondly, the CDS engine adapter, a service-oriented architecture (SOA)-based design, can provide a Web application programming interface for CPOE to invoke on demand the thematic CDS module with few configurations. It can also reduce the change in the original CPOE.

Moreover, as described in the Methods section (“Extension of Decision Support Function”), we can add EHR adapters to retrieve other EHR repositories not restricted in PharmaCloud. As increasing numbers of EHRs gradually become available, our CDS engine approach can rapidly meet the changing requirements of CDSS to provide more complete medical histories and ensure added safety. Other studies also indicated that the future adoption of CDS would ideally be modularized [33] with SOA design pattern [29] to account for the ever-changing medical knowledge. Thus, the innovative CDS engine framework can fulfill the trends in the field of medicine. The NHIA will continue to provide additional medical records, such as laboratory test results, surgeries, dental procedures, controlled drug management, and medicine allergies. With a more complete EHR, future iterations of the CDS engine would focus on the integration of more EHR repositories and designing of more CDS theme modules (ie, drug-drug interactions, dose calculations, pregnancy medication reminders, and allergy reminders) to extend CDS coverage to the health care facilities.

Electronic health records are generally recognized as key components of CDSSs [34]. Integrating EHRs with CDS functions is likely to become a widespread trend [32]. Many countries have taken steps to develop relevant infrastructures and regulations and provide incentive policies to facilitate the adoption and integration of EHR systems [35-37]. However, few studies have discussed that the CPOE framework automatically invokes the centralized national EHR for decision support when physicians prescribe medications. In Australia, a well-established nationalized EHR repository, My Health Record, primarily allows residents to maintain and share their health records with their general physicians or health care facilities. The Australian Digital Health Agency is currently investigating the secondary use of My Health Record [38], and our CDS engine application scenarios can be a reference for other countries who own centralized EHR repositories and are seeking secondary use applications.

Our study showed that 24.59% of patients signed the PharmaCloud consent form to allow their physicians to access their medication records. In our context, direct CDS engine users include authorized physicians, not patients. However, only after the patients sign the PharmaCloud consent form can the physicians access the patients’ medication histories in the PharmaCloud from their CPOE system. The lack of patient participation in the PharmaCloud system would increase the difficulty associated with the efficient implementation of protections against duplicate medication prescription across health care facilities. In Australia, My Health Record was originally adopted with opt-in consent; however, because the uptake rate remained low, two trial areas adopted opt-out consent until 2016 to increase the uptake rate. As a result, the uptake rates were obviously higher in these areas than in the non-trial areas [39-41]. Based on the implementation of the My Health Record system, we suggest that NHIA should consider adopting an opt-out rather than opt-in approach to increase the PharmaCloud usage for the CDS engine to provide a more comprehensive CDS support in health care facilities. Although an opt-out system is likely to increase the use of PharmaCloud, there are many complex factors and different national conditions that could affect patients’ participation in an innovative information system [42,43] and further research is needed to assess such factors.

### Limitations

The system proposed in this study has certain limitations. Firstly, the PharmaCloud batch mode requires patient consent to access his or her medication history prior to their hospital visit. In our approach, the PharmaCloud system provided only a batch download mode for the CDS engine to retrieve patient medication history, and the CDS engine local repository was updated once daily, usually at midnight. Therefore, it was not possible to use the CDS engine for checking prescriptions of walk-in patients. A previous study indicated that walk-in patients represent approximately 44% of total patients [44]. Thus, for such patients, physicians can only use a Web browser to access the PharmaCloud system to manually check the patient’s medication history. Secondly, due to our PharmaCloud consent rate of only 24.59%, the potential duplicate medication detection rate may have been underestimated. Finally, the CDS engine was adopted in a single teaching hospital; thus, the impact of adopting the CDS engine cannot be generalized to other hospitals at a national or international level.

### Conclusion

In this study, we developed a modularized CDS engine to access a national medication repository, the PharmaCloud, for detection of duplicate medication across health care facilities in Taiwan. Because of the modularized design, the CDS engine could easily extend functions for detection of ADR events when more and more EHR systems are adopted. Moreover, the CDS engine can retrieve more updated and completed patients’ medication histories in the PharmaCloud, so it can have better performance for detection of duplicate medication.

Although our CDS engine approach could enhance medication safety, it would make encounter time longer. Fortunately, this problem can be mitigated by careful evaluation of adopted solutions for implementation of the CDS engine.

Because the PharmaCloud system provided batch download mode only for the CDS engine to retrieve patients’ medication history, the CDS engine local repository could not be updated in a timely manner. Thus, the CDS engine might not be able to provide walk-in patients with protection from duplicate medication. To tackle this problem, we suggest PharmaCloud should consider the opt-out consent policy to increase the usability of the CDS engine to provide more comprehensive CDS support in health care facilities.

## Abbreviations

ADR: adverse drug reaction
ATC: Anatomical Therapeutic Chemical
CDS: clinical decision support
CDSS: clinical decision support system
CPOE: computerized physician order entry
EHR: electronic health record
NHI: National Health Insurance
NHIA: National Health Insurance Administration
SOA: service-oriented architecture
VPN: virtual private network
