# Authorship Correction: A Clinical Decision Support Engine Based on a National Medication Repository for the Detection of Potential Duplicate Medications: Design and Evaluation

**DOI:** 10.2196/15063

**Published:** 2019-07-05

**Authors:** Cheng-Yi Yang, Yu-Sheng Lo, Ray-Jade Chen, Chien-Tsai Liu

**Affiliations:** 1 Graduate Institute of Biomedical Informatics College of Medical Science and Technology Taipei Medical University Taipei Taiwan; 2 Department of Medical Informatics Industrial Technology Research Institute Hsinchu Taiwan; 3 Department of Surgery School of Medicine College of Medicine, Taipei Medical University Taipei Taiwan; 4 Taipei Medical University Hospital Taipei Taiwan

The authors of “A Clinical Decision Support Engine Based on a National Medication Repository for the Detection of Potential Duplicate Medications: Design and Evaluation” (JMIR Med Inform 2018;6(1):e6) made an error when designating equal contribution of authors. Both Cheng-Yi Yang and Yu-Sheng Lo should have been designated as equal contributors on this article.

The correction will appear in the online version of the paper on the JMIR website on July 5, 2019, together with the publication of this correction notice. Because this was made after submission to PubMed, PubMed Central, and other full-text repositories, the corrected article also has been resubmitted to those repositories.

